# Development and validation of a predictive nomogram for preoperative deep vein thrombosis (DVT) in isolated calcaneal fracture

**DOI:** 10.1038/s41598-022-10002-8

**Published:** 2022-04-08

**Authors:** Xinqun Cheng, Xiang Lei, Haifeng Wu, Hong Luo, Xiaorui Fu, Yicheng Gao, Xinhui Wang, Yanbin Zhu, Jincheng Yan

**Affiliations:** 1 Department of Orthopaedic Surgery, The 3rd Hospital of Hebei Medical University, Shijiazhuang, 050051 Hebei People’s Republic of China; 2 Orthopaedic Institution of Hebei Province, Shijiazhuang, 050051 Hebei People’s Republic of China; 3 Key Laboratory of Biomechanics of Hebei Province, Shijiazhuang, 050051 Hebei People’s Republic of China

**Keywords:** Biomarkers, Medical research, Risk factors

## Abstract

The fact that most of the patients with preoperative DVTs after calcaneal fractures are asymptomatic brought challenges to the early intervention, and periodic imaging examinations aggravated the financial burden of the patients in preoperative detumescence period. This study aimed to use routine clinical data, obtained from the database of Surgical Site Infection in Orthopaedic Surgery (SSIOS), to construct and validate a nomogram for predicting preoperative DVT risk in patients with isolated calcaneal fracture. The nomogram was established base on 7 predictors independently related to preoperative DVT. The performance of the model was tested by concordance index (C-index), receiver operating characteristic (ROC) curve, calibration curve, and decision curve analysis (DCA), and the results were furtherly verified internally and externally. 952 patients were enrolled in this study, of which 711 were used as the training set. The AUC of the nomogram was 0.870 in the training set and 0.905 in the validation set. After internal verification, the modified C-index was 0.846. Calibration curve and decision curve analysis both performed well in the training set and validation set. In short, we constructed a nomogram for predicting preoperative DVT risk in patients with isolated calcaneal fracture and verified its accuracy and clinical practicability.

## Introduction

Venous thromboembolism (VTE) has been the third most common cause of vascular mortality worldwide, which includes deep-vein thrombosis (DVT) and pulmonary embolism (PE)^1^. As one commonly seen injury, the calcaneal fracture is reported to be complicated by DVT in 3.1–12% of patients before they are surgically treated and almost all of the DVTs are asymptomatic^2,3^. Thus, it is difficult for early diagnosis and prompt targeted intervention. To improve the diagnostics ability of DVT in newly admitted patients, the development of early and efficient detection methods has been consistently the topic in scientific researches and clinical practice.

It is possible to address the issue by means of establishing a predictive model based on the medical condition surveys and hematological examinations on admission. Recently, Peng et al. successfully developed and prospectively validated a stratified risk model based on routine clinical data for predicting lower extremity DVT in patients with multiple trauma, and by comparison with TESS scores, this model showed excellent accuracy^4^. By far, there are several clinical scales for predicting thromboembolism, but there exist some controversial results regarding their effectiveness and validity. For example, the Wells score is advantageous in using some comprehensive variables to predict the possibility of developing DVTs^5^ but may be limited by dependence on subjective judgment and poor specificity, which thus is inapplicable to patients with acute trauma. Another commonly used tool, the Caprini's risk assessment model, which includes more than 30 risk factors in various aspects with an improved comprehensiveness and objectivity^6^, however, seems too cumbersome and complex to allow for a rapid clinical diagnosis. Furthermore, none of these thrombus prediction models is specific to trauma fields, not to mention the various fracture types on basis of locations, degree of displacement, comminution, and extent of involvement. Currently, there have been several studies on the identification of the risk factors for DVTs after foot and ankle trauma^3,7^, but they failed to translate the findings into practical DVT risk scales or predictive models. Indeed, in clinical practice, the role of these identified factors seem not to catch sufficient attention from clinicians, mainly due to that they were viewed in isolation. Therefore, it is almost impossible to use these “isolated” risk factors to determine the “intuitive” and “real” possibility that one patient develops a DVT.

Given the above, in this study, we aim to identify the risk factors associated with preoperative DVT in patients with isolated calcaneal fracture and, based on which, try to develop and validate an intuitive nomogram model to predict the DVTs risk to improve perioperative management for this acute trauma.

## Materials and methods

### General information

Data were derived from the database of Surgical Site Infection in Orthopaedic Surgery (SSIOS). The SSIOS database is a prospectively manually maintained database of all the data on hospitalized patients who experience orthopedic surgeries in the 3rd Hospital of Hebei Medical University. In this study, the clinical data of patients with calcaneal fractures treated surgically at our hospital from January 2016 to December 2019 were retrospectively analyzed to develop the model. The exclusion criteria were age under 18 years old, lack of preoperative duplex ultrasonography (DUS) examination, use of antithrombotic drugs (such as low molecular weight heparin, aspirin, etc.) three months prior to injury, multiple injuries, bilateral calcaneal fractures, open or pathological calcaneal fractures, and old fracture (≥ 21 days after injury). In addition, the clinical data of patients admitted from January 2020 to June 2021 was collected for external validation. The retrospective study was performed in accordance with the guideline of Strengthening the Reporting of Cohort Studies in Surgery (STROCSS) and complied with the principles of the Helsinki Declaration. The ethics committee of the 3rd Hospital of Hebei Medical University approved this study and all participants had signed an informed consent for the possible use of their clinical data for research purposes. According to the requirements for developing a clinical prediction model, the sample size should be at least 10 times the number of variables^8^. In our study there were 43 variables, thus the sample size should be at least 430, and therefore our sample size is adequate. In addition, as an exploration investigation, we did not specify a certain variable as primary outcome, so the method used in this study described as the previous study^8^ is more appropriate, which is different from the traditional one.

### Data collection

The clinical data of all patients were from 4 aspects, involving demographics, chronic comorbidities, injury-concerned data, and laboratory biomarkers. The demographics included patients’ gender, age, Living place, and the calculated body mass index (BMI). The comorbidities included alcohol drinking, smoking, Previous operation, History of allergy, hypertension, heart disease, and diabetes mellitus. Injury-concerned data comprised the mechanism of injury, fracture type according to the AO/OTA classification system, the time from injury to admission, and the time from injury to DUS examinations. The laboratory biomarkers included the count of red blood cell (RBC), neutrophil (NEU), lymphocyte (LYM), white blood cell (WBC), platelet (PLT); the levels of D-dimer, high-sensitivity C-reactive protein (HCRP), total protein (TP), albumin (ALB), fasting blood glucose (FBG), hemoglobin (HGB), hematocrit (HCT), alanine transaminase (ALT), aspartate transaminase (AST), alkaline phosphatase (ALP), antithrombin III (AT III), total cholesterol (TC), triglyceride (TG), high-density lipoprotein cholesterol (HDL-C), low-density lipoprotein cholesterol (LDL-C), very-low-density lipoprotein (VLDL), the platelet-to-hemoglobin ratio (PHR), the neutrophil-to-lymphocyte ratio (NLR), Sodium, fibrinogen (FIB), prothrombin time (PT), thrombin time (TT) and activated partial thromboplastin time (APTT). If the patient had undergone multiple hematology tests prior to the diagnosis of DVT, we selected the one closest to examination.

### Diagnosis and management of DVT

Duplex ultrasonography (DUS) examination was employed to clarify the presence of DVT. The criteria for the diagnosis of DVT were incompressibility of the vein, obstruction or filling defect of the lumen, absence of respiratory vibration in the venous segment above the knee, and insufficient flow augmentation to calf or foot after compression. The scanning range included all deep veins of both lower extremities from the inguinal ligament to the ankle and the DVTs located in the intermuscular vein (such as gastrocnemius vein) were excluded in this study for its relatively less clinical significance. Trauma patients in our hospital are routinely examined by DUS within 24 h of admission and get reviewed every 3 to 7 days when waiting for the operation. According to the standard treatment plan for patients with lower limb fractures in our hospital, all patients are required to elevate the affected extremity from admission. For patients with positive DUS results, the therapeutic doses of anticoagulant drugs (eg. enoxaparin sodium injection, 100 AxaIU/kg, twice daily) were prescribed, and for patients at high risk of thrombosis, such as the elderly, we would inject prophylactic doses of blood-thinning drugs (eg. enoxaparin sodium injection, 4000 AxaIU, once daily). For patients who need to wait a long time for surgery, we usually instruct patients to drink more water and move the affected limb appropriately, and the intermittent pneumatic pressure pumps were used to drive the blood circulation of lower extremities to prevent thrombosis.

### Statistical analysis

SPSS version 26.0 (IBM Corp, Armonk, NY, USA) was used for data analysis. Continuous data were presented as mean ± standard deviation (SD) when in normal distribution, otherwise as otherwise as the median and inter-quartile range (IQR). Kolmogorov–Smirnov test was performed to evaluate the normality of the continuous variables, and the Student t test or Mann–Whitney test was used for normally or non-normally distributed data according to the results. Categorical variables were expressed as numbers and percentages (%), and analyzed by chi-square or Fisher's exact test.

Plasma D-dimer and HCRP can reflect the development of DVT, but their elevated levels can also be affected by other factors, such as inflammation, so we decided to use the Youden-index to determine the optimal cut-off point. The same method was applied to determine the demarcation point of NLR and PHR.

All variables with *P* < 0.05 in univariate analysis were substituted into multivariate logistics regression analysis and screened by the backward stepwise regression method to identify the independent risk factors of DVT. The selected predictors were entered into R software (Version 3.6.5, R Foundation for Statistical Computing, Vienna, Austria) for further analysis, and the "rms" package was used to construct the nomogram. Conformance index (C-index), receiver operating characteristic (ROC) curve, calibration curve, and decision curve analysis (DCA) were used to evaluate the predictive power and application value of the model. The value of the C-index, ranged from 0.5 to 1.0, and the area under the receiver operating characteristic curve (AUC) were positively correlated with the discriminant ability and predictive accuracy of the nomogram. The calibration curve was used to evaluate the agreement between diagnosed thrombosis and predicted thrombosis, in which the higher the overlap between the predicted curve and the ideal curve, the better the consistency between the predicted probability and the true probability. The clinical application value of the nomogram model was evaluated by DCA, which was based on threshold probabilities and the net benefit. Finally, to further verify the prediction performance of the model, we used the Bootstrap method for internal verification, and calculate the modified C-index through 1000 repeated sampling. Then we employed the validation set for further external verification and compared the performances of ROC curves, calibration curves, and DCA of the nomogram in the training and validation cohorts. Significance levels were set at 0.05 for all analyses.

## Results

### Patient baseline data

**Figure 1:**
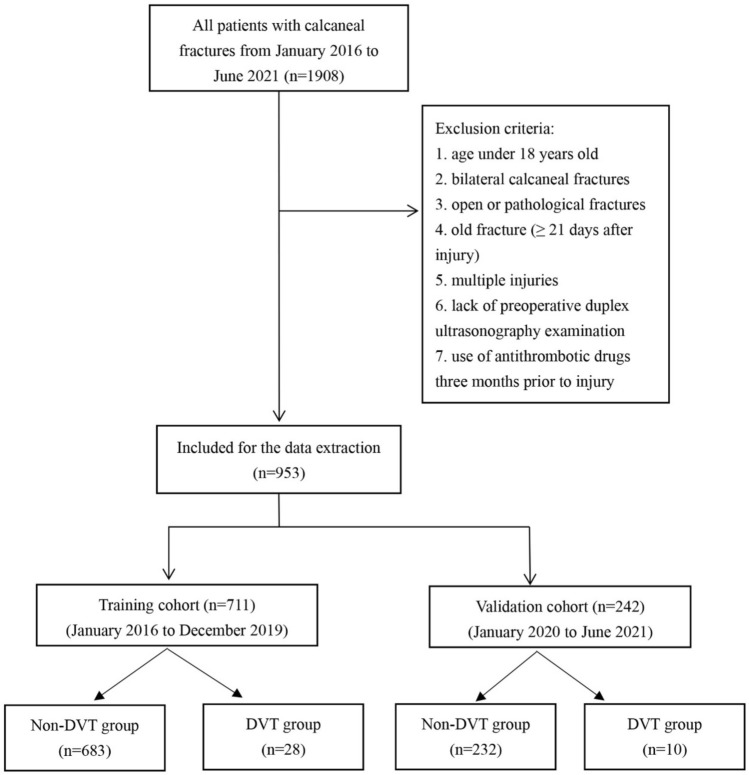
Patients selection flowchart.

According to the exclusion criteria, we selected 952 clinical data from the information of 1908 in-hospital patients with calcaneal fractures for this study, of which 711 were included to the training cohort (Fig. 1). Among the training set, 651 were males and 60 were females, with a mean age of 42.3 years (SD, 11.1 years), and 93.7% (666/711) were patients under 60 years old. As to the cause of injury, 70.5% (480) of patients were admitted due to falling from a height. In terms of fracture type, patients with type C fractures were in the majority (64.8%). The mean time from injury to admission was 1.6 days; The mean time from injury to DUS examination was 2.0 days. According to the results of ultrasonic examination, after excluding the blood clot located in the intermuscular vein, 28 patients were identified as having pre-operative DVT, representing a prevalence of 3.93%, and all of the DVTs patients were asymptomatic. The baseline characteristics of the training set were shown in Table 1.

**Table 1:** Univariate analysis of variables with interest between DVT and non-DVT patients.

Variables	Patients without DVT (*n* = 683)	Patients with DVT (*n* = 28)	*P*
Gender (males)	625 (91.5%)	26 (92.9%)	0.801
Age	43.0 (33.0, 51.0)	46.0 (34.3, 58.0)	0.138
**BMI**			0.788
< 18.5	7 (1.0%)	0 (0.0%)	
18.5–23.9	223 (32.7%)	11 (39.3%)	
24.0–27.9	347 (50.8%)	14 (50.0%)	
≥ 28.0	106 (15.5%)	3 (10.7%)	
Hypertension	48 (7.0%)	1 (3.6%)	0.479
Diabetes mellitus	17 (2.5%)	0 (0.0%)	0.398
Heart disease	6 (0.9%)	0 (0.0%)	0.618
Current smoking	123 (18.0%)	8 (28.6%)	0.158
Alcohol consumption	63 (9.2%)	5 (17.9%)	0.128
Living place (rural)	287 (42.0%)	11 (39.3%)	0.774
Previous operation	25 (3.7%)	1 (3.6%)	0.980
History of allergy	82 (12.0%)	3 (10.7%)	0.836
Mechanism (high energy)	462 (67.6%)	18 (64.3%)	0.710
**Fracture classification**			0.634
Type A	88 (12.9%)	2 (7.1%)	
Type B	154 (22.5%)	6 (21.4%)	
Type C	441 (64.6%)	20 (71.4%)	
Time from injury to admission (days)	1.0 (0.5,3.0)	4.0 (2.3,6.8)	< 0.001*
Time from injury to DUS (days)	2.0 (1.0,5.0)	5.0 (3.3,7.0)	< 0.001*
RBC (< lower limitation)	106 (15.5%)	17 (60.7%)	< 0.001*
HCT (< lower limitation)	210 (30.7%)	22 (78.6%)	< 0.001*
HGB (< lower limitation)	55 (8.1%)	11 (39.3%)	< 0.001*
PLT (> 300 × 10^9^/L)	64 (9.4%)	3 (10.7%)	0.811
PHR (> 1.62)	256 (37.5%)	18 (64.3%)	0.004*
WBC (> 10 × 10^9^/L)	180 (26.4%)	8 (28.6%)	0.794
NEU (> 6.3 × 10^9^/L)	291 (42.6%)	14 (50%)	0.438
LYM (< 1.8 × 10^9^/L)	453 (66.3%)	20 (71.4%)	0.575
NLR (> 3.09)	430 (63.0%)	25 (89.3%)	0.004*
TP (< 60 g/L)	76 (11.1%)	12 (42.9%)	< 0.001*
**ALB**	42.42 ± 3.95	39.64 ± 3.65	< 0.001*
< 35 g/L	22 (3.2%)	2 (7.1%)	0.260
**FBG**	5.4 (5.0, 5.9)	6.0 (5.0, 6.7)	0.043*
> 6.1 mmol/L	132 (19.3%)	11 (39.3%)	0.010*
HCRP (> 23.34 mg/L)	216 (31.6%)	15 (53.6%)	0.015*
Sodium (< 135 mmol/L)	33 (4.8%)	2 (7.1%)	0.580
TG (> 1.7 mmol/L)	119 (17.4%)	5 (17.9%)	0.953
TC (> 5.2 mmol/L)	86 (12.6%)	1 (3.6%)	0.153
HDL-C (< 1.1 mmol/L)	242 (35.4%)	18 (64.3%)	0.002*
LDL-C (> 3.37 mmol/L)	106 (15.5%)	2 (7.1%)	0.226
VLDL (> 0.78 mmol/L)	115 (16.8%)	5 (17.9%)	0.888
ALT (> 50U/L)	110 (16.1%)	7 (25.0%)	0.213
AST (> 40U/L)	48 (7.0%)	3 (10.7%)	0.459
ALP (> 135U/L)	6 (0.9%)	0 (0.0%)	0.618
D-dimer (> 1.92 mg/L)	138 (20.2%)	11 (39.3%)	0.015*
AT III (< 80%)	33 (4.8%)	5 (17.9%)	0.003*
PT (> 12.5 s)	114 (16.7%)	6 (21.4%)	0.512
**APTT (28-42 s)**			0.696
< 28	173 (25.3%)	9 (32.1%)	
> 42	2 (0.3%)	0 (0.0%)	
TT (12-17 s)			0.489
< 12	21 (3.1%)	2 (7.1%)	
> 17	28 (4.1%)	1 (3.6%)	
**FIB (2–4.4 g/L)**			0.703
< 2	13 (1.9%)	1 (3.6%)	
> 4.4	101 (14.8%)	3 (10.7%)	

*BMI* Body mass index, *RBC* red blood cell, reference range: Female, 3.5–5.0 × 10^12^/L; males, 4.0–5.5 × 10^12^/L; *HCT* hematocrit, reference range: Females, 35–45%; males, 40–50%; *HGB* hemoglobin, reference range: Females, 110–150 g/L; males, 120–160 g/L; *PLT* platelet, *PHR* the platelet-to-hemoglobin ratio, *WBC* white blood cell, *NEU* neutrophil, *LYM* lymphocyte, *NLR* the neutrophil-to-lymphocyte ratio, *TP* total protein, *ALB* albumin, *FBG* fasting blood glucose, *HCRP* high-sensitivity C-reactive protein, *TG* triglyceride, *TC* total cholesterol, *HDL-C* high-density lipoprotein cholesterol, *LDL-C* low-density lipoprotein cholesterol, *VLDL* very low-density lipoprotein, *ALT* alanine transaminase, *AST* aspartate transaminase, *ALP* alkaline phosphatase, *AT III* antithrombin III, *PT* prothrombin time, *APTT* activated partial thromboplastin time, *TT* thrombin time, *FIB* fibrinogen.

*Statistical significance.

### Univariate and multivariate analysis

Before univariate analysis, we used the Youden-index to determine the optimal cutoff values for the following categorical variables: D-dimer (1.92 mg/L), HCRP (23.34 mg/L), NLR (3.09), PHR (1.62). In univariate analysis, we identified 15 potential predictors from continuous and classified variables (Table 1). In multivariate analysis, the backward stepwise regression method was used to screen these predictive factors, and 7 variables were finally retained. The multivariate logistics regression analysis demonstrated that D-dimer (> 1.92 mg/L, odds ratio (OR) = 2.76, 95% confidence interval (CI) = 1.06–7.20, *P* = 0.038), ALB (OR = 0.85, 95%CI = 0.76–0.95, *P* = 0.005), GLU (> 6.1 mmol/L, OR = 3.04, 95%CI = 1.22–7.61, *P* = 0.017), time from injury to DUS (OR = 1.42, 95%CI = 1.27–1.60, *P* < 0.001) , AT III (< 80%, OR = 6.89, 95%CI = 1.89–25.11, *P* = 0.003), NLR (> 3.09, OR = 6.02, 95%CI = 1.34–27.04, *P* = 0.024) and PHR (> 1.62, OR = 2.57, 95%CI = 1.01–6.56, *P* = 0.042) were independent risk factors for preoperative DVT in patients after isolated calcaneal fracture (Table 2).

**Table 2:** Multivariate analyses of the risk factors related to preoperative DVTs following isolated calcaneal fracture.

Variables	OR and 95%CI	*P* value
FBG (> 6.1 mmol/L)	3.04 (1.22–7.61)	0.017
ALB (g/L)	0.85 (0.76–0.95)	0.005
D-dimer (> 1.92 mg/L)	2.76 (1.06–7.20)	0.038
AT III (< 80%)	6.89 (1.89–25.11)	0.003
PHR (> 1.62)	2.57 (1.01–6.56)	0.042
NLR (> 3.09)	6.02 (1.34–27.04)	0.019
Delay from injury to DUS (days)	1.42 (1.27–1.60)	< 0.001

*OR* odd ratio, *CI* confidence interval, *FBG* fasting blood glucose, *ALB* albumin, *AT III* antithrombin III, *PHR* the platelet-to-hemoglobin ratio, *NLR* the neutrophil-to-lymphocyte ratio.

### Development and validation of a predictive nomogram

**Figure 2:**
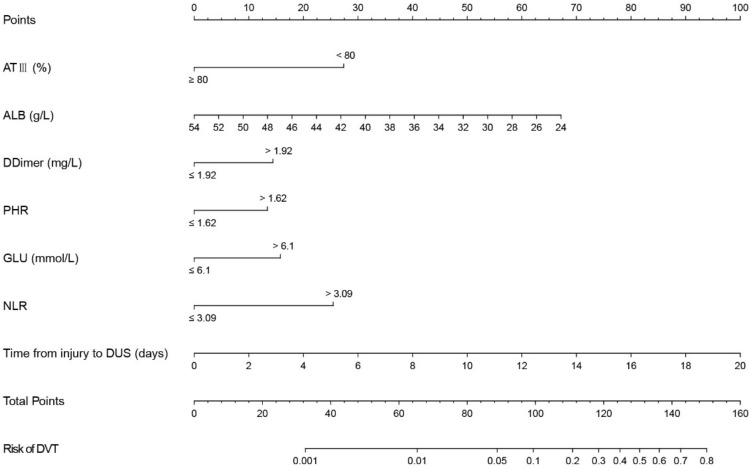
Nomogram for predicting preoperative DVTs in patients with isolated calcaneal fracture. The sum of the scores of each predictor (D-dimer, albumin, blood glucose, time from injury to DUS, NLR, PHR, and AT III) corresponds to the risk of DVT.

**Figure 3:**
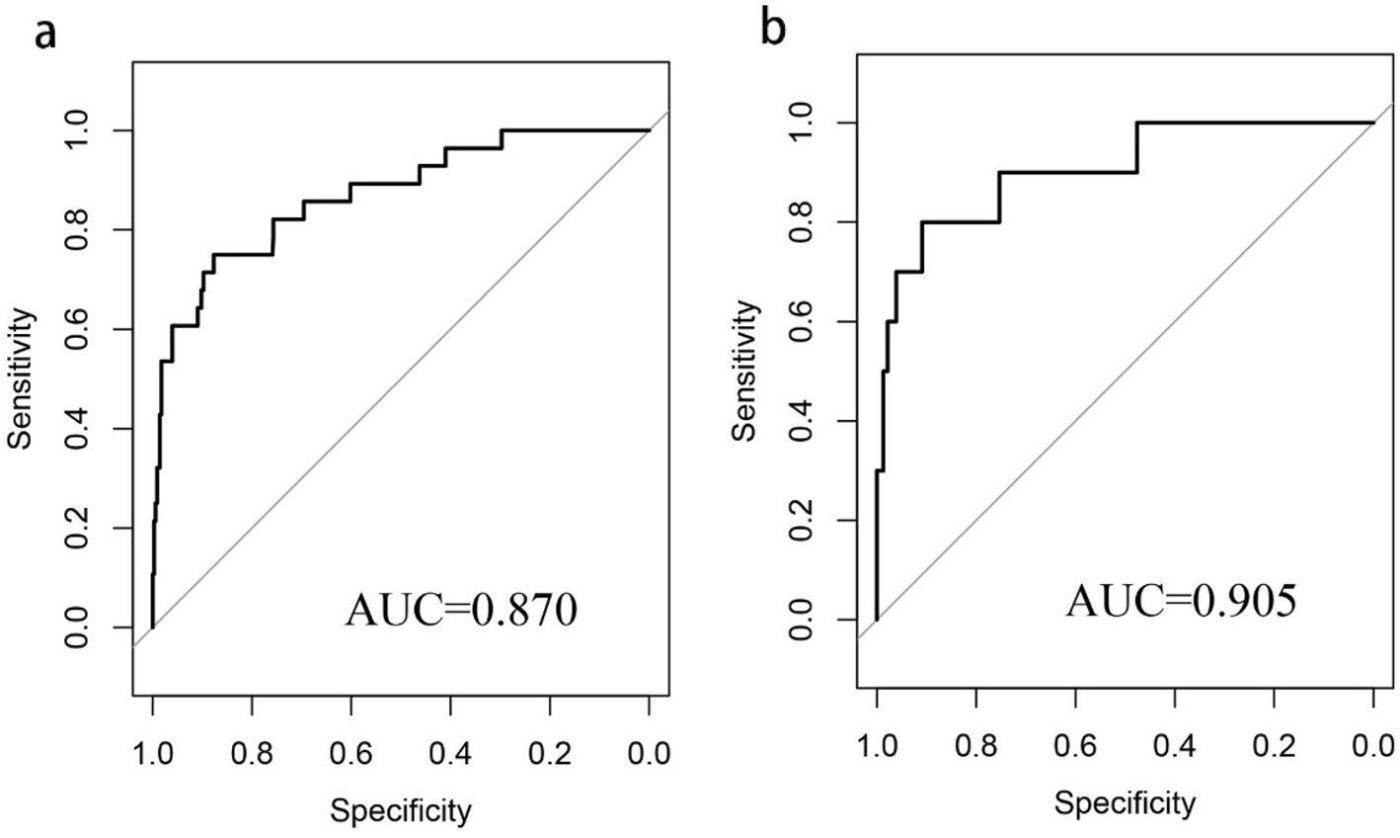
Comparison of the receiver operating characteristic curve (ROC) of the nomogram in the training cohort (**a**) and the validation cohort (**b**). The area under curve (AUC) were positively correlated with the predictive accuracy of the nomogram.

**Figure 4:**
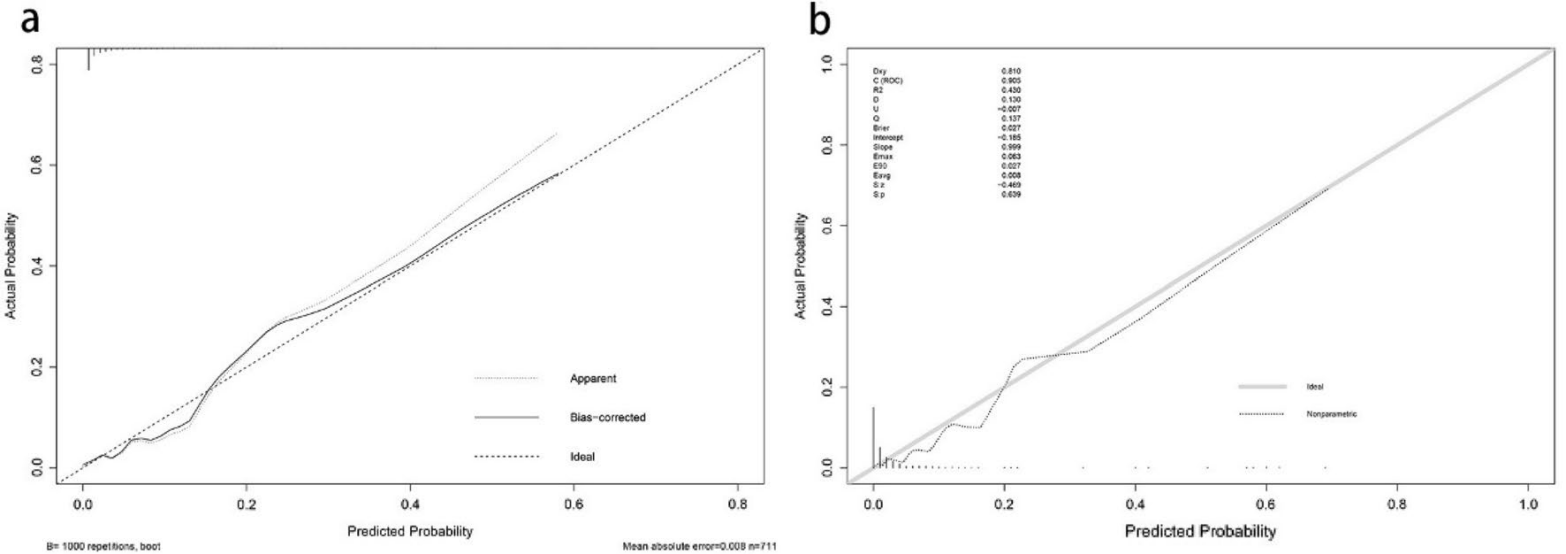
Comparison of the calibration curves of the nomogram in the training cohort (**a**) and the validation cohort (**b**). In the calibration curve, the higher the overlap between the predicted curve and the ideal curve, the better the consistency between the predicted probability and the true probability.

**Figure 5:**
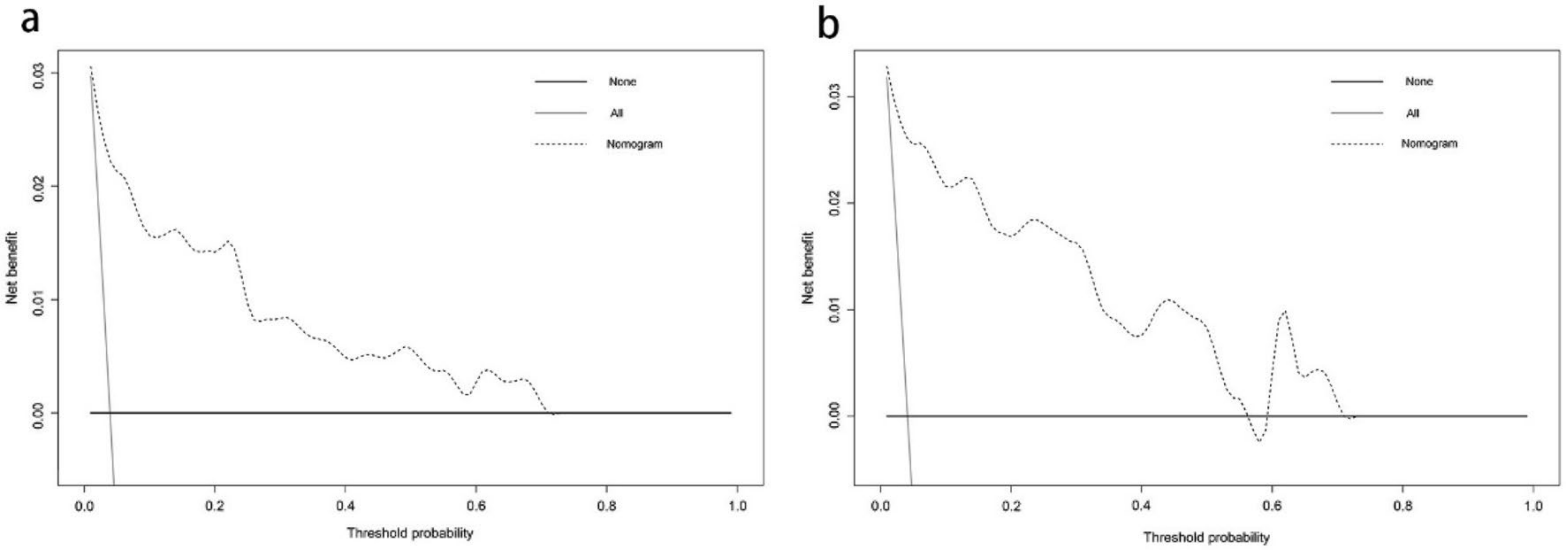
Comparison of the decision curve analyses (DCA) of the nomogram in the training cohort (**a**) and the validation cohort (**b**). The X-axis represents the threshold probability and the Y-axis represents the net benefit. The dotted line represents the nomogram, The black line assumed that no patient has DVT before surgery, while the gray line represented the assumption that all patients have preoperative DVT. The range of threshold probabilities representing positive net benefit is obtained according to the corresponding points of the intersection of the dotted line with the black line and grey line on the X-axis.

We substituted these 7 predictors into a binary logistic regression analysis and transformed the results into a nomogram that could be used to predict the risk of preoperative DVT (Fig. 2), and furtherly evaluated the performance and reliability of the nomogram. The AUC for the predictive model was 0.870 (95%CI: 0.795–0.945) (Fig. 3), with a sensitivity of 75.0% and specificity of 87.7%, indicating that the model had a strong discriminatory ability. The C-index for the nomogram was 0.870 (95%CI: 0.797–0.944) and the modified value was 0.846 after 1000 bootstrap verifications, which suggested the good refinement of the model. In addition, the calibration curve (Fig. 4) showed that the predicted probability of DVT in trauma patients by nomogram is in good agreement with the actual probability. DCA of nomogram showed that compared to no intervention, using DVT prediction nomogram can bring positive net benefit when the threshold probability was in the range of 2–72% (Fig. 5). Finally, the performances of ROC (AUC: 0.870 vs. 0.905), the calibration curve, and the decision curve analysis showed good consistency in the comparison of the training cohort and validation cohort.

### Instructions for the use of the predictive nomogram

As shown in Fig. 2, we established a nomogram containing 7 covariates. When using the nomogram, first, the ATIII (%) of the subject was positioned on the corresponding variable axis; next, draw a vertical line to the “Points” axis to obtain the corresponding score (when ATIII < 80%, the corresponding score was 27). Repeat the above process to get the scores of each covariable, and finally add up to get the total score. Find the corresponding point of the total score on the “Total Points” axis and make a vertical line to the “Risk of DVT” axis to get the possibility of developing DVT.

### Ethics approval and consent to participate

The retrospective study was authorized by the ethics committee of the 3rd Hospital of Hebei Medical University and the patients have given their informed consent.

## Discussion

To our knowledge, this is the first study to establish and validate a nomogram to predict the risk of preoperative DVT in patients with isolated calcaneal fracture. The results showed that the incidence of DVT was 3.9% and in multifactorial analysis, D-dimer, albumin, blood glucose, time from injury to DUS, NLR, PHR, and AT III were identified as independent risk factors for thrombosis. The nomogram based on these 7 factors showed good performance with an AUC value of 0.870 (> 0.8, 95% CI: 0.795–0.945), a sensitivity of 75.0%, and a specificity of 87.7%. More importantly, the results of both internal validation and external validation proved the good consistency of this model.

Elevated D-dimer levels, hypoproteinemia, and hyperglycemia are common risk factors for thrombosis in traumatic patients, and their correlation with venous thrombosis has been widely discussed in works of literature^9–11^. Albumin and blood glucose are potentially adjustable nutritional or metabolic indicators, and recent studies have confirmed that their abnormal status was closely associated with the incidence of postoperative incisional complications in patients with calcaneal fractures, so positive correction of these indexes may provide clinical value to prevent thrombosis and improve prognosis. In addition to hypercoagulability status or thrombosis, D-dimer level is also affected by other factors, such as advanced age, tumor, and inflammation^12,13^, making D-dimer less specific in the diagnosis of DVT. Consequently, we used the Youden-index to determine the optimal cut-off value of the D-dimer level (1.92 mg/L), which was significantly higher than the traditionally used one (0.5 mg/L). Compared with the traditional one, the adjusted value can significantly improve the specificity from 35.6 to 79.4%, which is helpful in excluding non-DVT patients. Although the sensitivity of D-dimer testing will be reduced (from 78.1 to 40.6%), this deficiency can be compensated by the combination of D-dimer and other risk factors^14,15^, as also presented in our study.

NLR, a hot hemocyte derivative index, reflects the status of systemic inflammatory/immune response, and its potential association with acute pulmonary embolism and the predictive value for early DVT has been confirmed by studies^16–18^. The internal mechanism might be related to the inflammatory cascade of cytokines and chemokines, which can promote the aggregation and increase of neutrophils^19^. Recently, this inflammatory index was demonstrated to be associated with the risk of perioperative DVT in patients treated with major orthopedic surgeries^20–22^. Seo et al. retrospectively studied the clinical data from 264 patients undergoing total knee arthroplasty and found that preoperative elevated NLR level is an independent risk factor for postoperative thrombotic risk^21^. Our study showed that NLR level above 3.09, when newly admitted, was independently associated with a 6.0-fold risk of preoperative DVT for patients with isolated calcaneal fracture.

PHR is a hematological indicator that reflects systemic inflammatory and nutrition status, and in this study, we found that PHR levels above 1.62 were independently related to a 2.6-fold increased risk of developing preoperative DVT. This could be explained by the fact that both anemia and thrombocytosis contribute to the development of vein thrombosis after acute trauma^23–25^, and this ratio further exaggerates this predictive effect. In the field of orthopedics, there are few studies on the association between PHR and complications following acute trauma, while in other fields, PHR was used as a useful preoperative indicator to estimate the risk of positive bone scintigraphy at renal cell carcinoma staging^26^ and to predict the prognosis of patients undergoing renal cell carcinoma surgery^27^. Therefore, further studies should be carried out to explore the potential predictive value of PHR for perioperative complications in patients with fractures, such as DVT, and surgeons should be aware of the clinical importance of controlling the modifiable factors as such and thus to correct preoperative anemia if necessary.

In this study, we found that patients with DVT waited significantly longer for DUS than those without DVT (5.3 days vs. 1.9 days), and multivariate analysis showed a 42% increased risk of DVT for each day of delay from injury to DUS examination. In this study, waiting a long time for DUS examination is mainly attributed to the delay from injury to admission (1.5 days vs. 4.7 days), which is very common in large tertiary hospitals with a high number of patients referred from the remote hospital, especially for patients with multiple injuries. In addition, due to the special condition of patients with calcaneal fractures, patients with swollen feet are often obliged to wait for 1–2 weeks to eliminate swelling before surgical intervention for aims to reduce the incidence of wound complications^28^. Therefore, according to our findings, early ultrasound examination, and anticoagulation if necessary are recommended for patients with delayed admission, and regular DVT screening and enhanced preoperative management for thrombosis prevention should be performed for patients in the swelling stage.

Nomograms, which can leverage the predictive value of each risk factor and visualize the final prediction results, have been widely used in studies of clinical predictive models^29–32^. Thus, we successfully established and validated a nomogram to facilitate clinicians' preoperative DVT risk assessment in newly admitted patients with calcaneal fractures. The seven predictors included in this nomogram were obtained from routinely collected clinical data and available within hours of admission. Nomograms can help clinicians quickly identify patients with a higher risk of DVT. When using the nomogram, clinicians only need to draw vertical lines according to the results of different variables to get the prediction probability of each variable, and finally calculate the sum to get the corresponding risk value. For patients at presumed higher risk, ultrasound examinations should be performed as soon as possible to determine the presence of DVT, and aggressive drug therapy should be administered to prevent the further development of thrombosis. For patients in the swelling stage, this model can help doctors monitor the risk of DVTs regularly, which can not only improve the clinical value of hematological examinations but also reduce the expense of regular imaging examinations.

The advantages of this study included the establishment of a nomogram and confirmation of its effectiveness via internal and external validation, and also identification of PHR as a promising and independent predictor for DVT. However, several limitations must be mentioned. Firstly, selection bias is inevitable due to the inherent characteristics of retrospective studies. Secondly, some variables that may be significantly related to the occurrence of DVT were not recorded or unmeasured, such as the duration of limb immobilization. Thirdly, the clinical data used to develop and verify the nomogram were single-center, and the findings should be treated cautiously when applied to different regions and groups. Prospective observational studies with multicenter data are needed to confirm the clinical applicability of the model.

## Conclusion

In summary, we identified that D-dimer, albumin, blood glucose, time from injury to examination, NLR, PHR, and AT III were significant predictors of preoperative DVT in patients with isolated calcaneal fracture. We convert this result into a nomogram to predict the risk of preoperative DVT. The nomogram showed great discriminative ability and clinical practicability. Furthermore, we carried out further internal and external verification of the model and obtained satisfactory results. Clinicians can employ the nomogram to identify the risk of preoperative DVT in hospitalized patients with calcaneal fractures so that early interventions can be made as necessary to prevent catastrophic consequences caused by the further development of thrombus.

## Data Availability

All the data used during the current study are available from the corresponding author on reasonable request.
